# Relationships between Severity of Internet Gaming Disorder, Severity of Problematic Social Media Use, Sleep Quality and Psychological Distress

**DOI:** 10.3390/ijerph17061879

**Published:** 2020-03-13

**Authors:** Hiu Yan Wong, Hoi Yi Mo, Marc N. Potenza, Mung Ni Monica Chan, Wai Man Lau, Tsz Kwan Chui, Amir H. Pakpour, Chung-Ying Lin

**Affiliations:** 1 Department of Rehabilitation Sciences, The Hong Kong Polytechnic University, Hung Hom, Hong Kong; anniehiuyan@gmail.com (H.Y.W.); elainemohoiyi@gmail.com (H.Y.M.); monicachan0710@gmail.com (M.N.M.C.); sunny313971048@gmail.com (W.M.L.); chuitszkwan1996@gmail.com (T.K.C.); 2 Departments of Psychiatry and Neuroscience and the Child Study Center, School of Medicine, Yale University, New Haven, CT 06511, USA; marc.potenza@yale.edu; 3 Connecticut Council on Problem Gambling, Wethersfield, CT 06109, USA; 4 Connecticut Mental Health Center, New Haven, CT 06519, USA; 5 Social Determinants of Health Research Center, Research Institute for Prevention of Non-Communicable Diseases, Qazvin University of Medical Sciences, Qazvin 3419759811, Iran; 6 Department of Nursing, School of Health and Welfare, Jönköping University, 55511 Jönköping, Sweden

**Keywords:** gaming, social media, behavior addiction, sleep quality, psychological distress

## Abstract

Internet gaming and social media use are prevalent and integral to many people’s lives. However, excessive engagement in either could lead to negative health impacts. This study aimed to investigate relationships between severities of internet gaming disorder (IGD) and problematic social media use (operationalized as social media addiction; SMA) with sleep quality and psychological distress among young adults. A cross-sectional study with snowball sampling was conducted among Hong Kong university students in 2019. All participants (*n* = 300; mean (SD) age = 20.89 (1.48); 122 males (40.67%)) responded to an online survey that included Chinese versions of the Internet Gaming Disorder Scale-Short Form (IGDS9-SF), Bergen Social Media Addiction Scale (BSMAS), Pittsburgh Sleep Quality Index (PSQI), and Depression Anxiety Stress Scales (DASS-21). Multiple linear regressions demonstrated that IGDS-SF9 scores demonstrated associations with psychological distress measures (standardized coefficient (β) = 0.295 for depression, 0.325 for anxiety, 0.339 for stress, all *p* < 0.001). BSMAS scores showed similar albeit numerically less robust associations (β = 0.235 for depression, *p* < 0.001; 0.219 for anxiety, *p* = 0.001; 0.262 for stress, *p* < 0.001). BSMAS scores demonstrated associations with poorer sleep quality (β = 0.292; *p* < 0.001) and IGDS9-SF scores (β = 0.157; *p* = 0.024) showed a significantly less robust association (*p* = 0.01 for comparing the two βs). These findings suggest that both severities of IGD and SMA associate with more psychological distress and poorer sleep quality, although the strengths of associations may differ.

## 1. Introduction

Serious health concerns have been linked to specific types and patterns of engagement in internet usage, leading to the concept of “internet addiction” [1,2]. The availability, accessibility and affordability of internet usage worldwide may lead to worsening health problems. According to the International Telecommunication Union (ITU) [3], global internet usage increased from 1990 million in 2010 to 3385 million in 2016. In Hong Kong and Macau, it is estimated that 23.3% of young adults aged 18 to 30 years experience problems with online gaming [4]. Therefore, problematic gaming is a significant concern for young adults in these Asian jurisdictions, and findings suggest similar concerns in other (American, European) countries. In addition to online gaming, internet-based social media platforms (e.g., YouTube, Snapchat, and Instagram) are frequently used, with nearly three fourths (71%) of young adults aged 18–24 years in the US using these media platforms doing so multiple times per day [5]. As such, many young adults may be vulnerable to experiencing Internet gaming and/or social media addictions.

Definitions for addictions have changed over time. Recently, the American Society of Addiction Medicine defined addiction as, “a treatable, chronic medical disease involving complex interactions among brain circuits, genetics, the environment, and an individual’s life experiences. People with addiction use substances or engage in behaviors that become compulsive and often continue despite harmful consequences” [6]. Indeed, *behavioral addictions* have been included in the latest edition of the *Diagnostic and Statistical Manual of Mental Disorders (DSM-5)*, with gambling disorder and internet gaming disorder (IGD) defined, with the latter as a provisional diagnostic entity [7]. Recently, *gaming disorder* has been added to the *International Classification of Diseases 11th Revision (ICD-11)* as a disorder due to addictive behaviors [8]. With poor control over repetitive, persistent, and dysfunctional behaviors, behavioral addictions can result in negative health consequences, including social relationship problems, impairments in functional roles and activities, sleep problems, and mental disorders [9]. Therefore, assisting people, especially young adults, in tackling behavioral addictions is important for healthcare providers.

Debates have focused on whether generalized internet addiction or specific internet addictions are diagnosable entities, and if the latter, which disorders constitute specific internet addictions [10,11]. In the current study, we focused on gaming and social media addictions given their potential impacts. Both social media addiction (SMA) and IGD have been associated with sleep disturbances, including sleep quality deterioration and reductions in sleep duration [12,13]. A potential reason for both SMA and IGD leading to sleep disturbances involves portable smartphones and other portable digital devices that may be brought to bed [13].

With portable smartphones and Wi-Fi access, internet (including of social media) may constitute an unstructured activity, one not limited in time and one that can displace time [14]. As such, SMA may lead to lack of sleep. Delayed bedtimes and late waking times may further lead to the rhythm desynchronization and impact school performance [15]. Other potential mechanisms in disrupting sleep may include psychological stimulation (i.e., excited mood due to use of social media) [16], light-emitting screens (i.e., light may suppress sleep-promoting hormones like melatonin that are typically elevated before bedtime) [17], and shortened sleep duration [18]. Similar considerations exist for online gaming. Gamers from different time zones often participate in multiplayers games [12]. Therefore, some gamers may avoid logging off or may arise in the middle of the night to continue gaming [19], leading to irregular or disorganized sleep-wake patterns. Individuals may become sleep-deprived in order to engage in virtual activities. Consistently, playing online games has been associated with longer sleep latencies and shorter total rapid eye movement (REM) sleep [20].

Apart from negative effects on sleep, SMA and IGD may impact psychological well-being, with associations with depression, anxiety, and stress [2,21]. “Fear of missing out” may keep individuals on social media sites and lead to stress and burnout [4,21]. Internet addiction, as a general concept, may lead to poor sleep quality and psychological problems [2,22,23,24]. Both SMA [25,26,27,28,29] and IGD [30,31,32] have been associated with poor psychological health. However, differential associations of IGD and SMA with health outcomes have been rarely examined and compared in the same studies. To our best knowledge, only one study has examined relationships between IGD/SMA and symptoms of psychiatric disorders (i.e., attention-deficit/hyperactivity disorder, obsessive- compulsive disorder, anxiety, and depression) [33] and another has examined relationships between IGD/SMA and psychological distress (i.e., anxiety, stress, and depression) [32]. Therefore, more research is needed. Specifically, the activity demands and behavioral patterns of the two addictions are different. As internet gaming and use of social media may have different activity demands and behavioral patterns, with gaming typically requiring more rapid reactions and sustained attention over a continuous time period than using social media use, IGD and SMA may differentially impact sleep quality and psychological distress.

The current preliminary study was designed to explore how severities of IGD and SMA relate to sleep quality and emotional distress in a population at risk of internet addiction (i.e., young adults) [34] in order to complement prior studies of primary and secondary school students [1,35]. We hypothesized that both severities of IGD and SMA would relate to poorer sleep quality and more emotional distress.

## 2. Materials and Methods

A cross-sectional study using convenience and snowball sampling was conducted among university students aged 18–24 in Hong Kong in 2019.

### 2.1. Participants and Procedure

Data were collected from April to June 2019 using *Google Form*. The inclusion criteria were: (1) currently studying as an undergraduate or a postgraduate in a university or college in Hong Kong; (2) able to understand written Chinese in traditional characters and spoken Chinese in Cantonese; (3) currently possessing a smartphone with Internet access. The exclusion criterion was having a self-reported diagnosis of psychiatric problems or neurological disease. Finally, 306 participants agreed to participate in this study and data from 300 were analyzed after removing data from those who were not eligible (the six participants were removed because they were not university students or not studying in Hong Kong).

An invitation message with an online link to the survey was sent to the existing social network of the present study’s Hong Kong researchers. The recipients were invited to complete an online survey and spread the message to their university peers. The message explained the present study’s objectives and the estimated time for completing the survey. Before survey commencement, a project information sheet explaining the study aims, survey structure, and confidentiality assurance was provided to the participants. Participants could enter the survey only after they provided an online consent form that expressed their willingness to participate. The survey included a background information sheet and the Chinese versions of the following questionnaires: the Internet Gaming Disorder Scale-Short Form (IGDS9-SF), Bergen Social Media Addiction Scale (BSMAS), Pittsburgh Sleep Quality Index (PSQI) and Depression Anxiety Stress Scales (DASS-21). The background information sheet included questions relating to age, gender, education level, height, weight, smoking history, monthly income, time spent on smartphone per day in the past month, time spent on internet gaming per day in the past month, and time spent on social media per day in the past month. Five university students completed the survey to help ensure the survey readability before recruiting study participants. The study was approved by the Human Subjects Ethics Subcommittee of The Hong Kong Polytechnic University (Ref No. HSEARS20190130006).

### 2.2. Instruments

#### 2.2.1. Internet Gaming Disorder Scale- Short Form (IGDS9-SF)

The 9-item IGDS9-SF assesses the severity of Internet Gaming Disorder (IGD) and its detrimental effects over a 12-month period [36,37,38,39,40,41,42]. The nine questions correspond to the nine DSM-5 criteria for IGD [7]. The items are rated using a 5-point Likert scale from 1 (*Never*) to 5 (*Very often*). A total score can be obtained by summing responses with a range from 9 to 45 points, with higher scores reflecting greater IGD severity. The cut-off score over 21 indicates problematic internet gaming [43]. A sample question is, “Do you feel the need to spend increasing amount of time engaged gaming in order to achieve satisfaction or pleasure?” The instrument has demonstrated validity and reliability for assessing IGD [36,37,38,39,40,41,42]. IGDS9-SF scores have been correlated with weekly time spent gaming (r = 0.325, *p* < 0.0001) and IGD-20 Test scores (r = 0.842, *p* < 0.0001), providing evidence for validity [44]. The IGDS9-SF has demonstrated high internal consistency (α = 0.87) and low floor or ceiling effects [44]. The IGDS9-SF has also been validated among Hong Kong participants [45,46]. The internal consistency of the IGDS9-SF in the present study was 0.91.

#### 2.2.2. The Bergen Social Media Addiction Scale (BSMAS)

The BSMAS is a self-report scale for assessing SMA severity [33,47,48,49]. It contains six items relating to six core addiction components (salience, mood modification, tolerance, withdrawal, conflict, and relapse) [50]. Each item examines the experience of using social media over the last year and is rated on a five-point Likert scale ranging from 1 (*very rarely*) to 5 (*very often*), producing a composite score ranging from 6 to 30. A sample question from the BSMAS is, “How often during the last year have you used social media so much that it has had a negative impact on your job/studies?” Higher BSMAS scores indicate greater SMA severity. A cut-off score over 19 indicates problematic social media use [51]. The BSMAS has satisfactory psychometric properties with high internal consistency (α = 0.819) and low floor and ceiling effects [34]. BSMAS scores correlated with IGDS9-SF scores and assessments of anxiety and depression [34]. The BSMAS has been previously validated among Hong Kong participants [45,46]. The internal consistency of the BSMAS in the present study was 0.83.

#### 2.2.3. Depression Anxiety Stress Scales (DASS-21)

The DASS-21 includes three self-reported subscales to measure negative emotional states, including depression, anxiety, and stress [52]. Each scale consists of seven items [52]. By using a four-point severity/ frequency scale, each participant is asked to rate the extent to the experiences relate to each negative emotion. The rating scale for each item is comprised of 0 *(did not apply to me at all*), 1 (applied *to me to some degree, or some of the time*), 2 *(applied to me to a considerable degree, or a good part of time*) and 3 *(applied to me very much, or most of the time*). A sample question from the DASS-21 depression subscale is, “I felt that I had nothing to look forward to.” A sample question from the DASS-21 anxiety subscale is, “I was aware of dryness of my mouth.” A sample question from the DASS-21 stress subscale is, “I found it hard to wind down.” The scores for depression, anxiety, and stress are summed, respectively, to generate scores in each domain [52]. The high internal consistency (α = 0.94, 0.88, and 0.93 for depression, anxiety, and stress, respectively) of the DASS-21 reflects its reliability [53]. The DASS-21 has been validated among Hong Kong participants [52]. The internal consistency of the DASS-21 in the present study was 0.88 (Depression domain), 0.83 (Anxiety domain), and 0.85 (Stress domain).

#### 2.2.4. The Pittsburgh Sleep Quality Index (PSQI)

The PSQI assesses sleep quality in clinical populations using nineteen questions that can be classified into seven domains that include subjective sleep quality (Q6), sleep latency (Q2 and Q5a), sleep duration (Q4), habitual sleep efficiency (Q1, Q3 and Q4), sleep disturbances (Q5b-Q5j), use of sleeping medication (Q7), and daytime dysfunction (Q8 and Q9) [54]. The items examine sleep quality and disturbances over a 1-month period. A sample question from the PSQI is, “How often have you had trouble sleeping because you have bad dreams?” Scores for each question range from 0 (*no difficulty*) to 3 (*severe difficulty*). The summed component scores generate a global score ranging from 0 to 21, where higher scores represent poorer sleep quality. A cutoff score of 5 for sleep impairment has been proposed for distinguishing good sleep and poor sleep [54]. PSQI scores have correlated with severity of Internet addiction [55]. The PQSI has demonstrated high internal consistency (α = 0.83). Both global scores (T1-T2 correlation coefficient = 0.85, *p* < 0.001) and component scores such as sleep latency (T1-T2 correlation coefficient = 0.84, *p* < 0.001) show high test-retest reliability [54]. The PSQI has been suggested as a good screening test for insomnia in college students, with a recommended cut-off ≥ 6 due to its high diagnostic accuracy [56]. The Chinese PSQI has been validated among general and clinical populations in Hong Kong [57,58].

### 2.3. Statistical Analysis

Statistical analysis was performed using Statistical Package for the Social Sciences (SPSS) version 24.0 (IBM Inc., Armonk, NY, USA). Pearson’s correlations were applied to examine the bivariate correlations between IGDS9-SF, BSMAS and PSQI scores and DASS-21 domains. Fisher’s *r*-to-*z* transformation on dependent samples (http://quantpsy.org/corrtest/corrtest2.htm) was used to examine for significant differences in magnitudes of bivariate correlations. The associations of severities of IGD and SMA and health outcomes were further analyzed using regression models, which controlled for potential confounders. Specifically, DASS-21 domain scores (i.e., depression, anxiety, and stress domains) and PSQI score were entered as dependent variables; IGDS9-SF and BSMAS were used as independent variables; age, gender, time spent on smartphones, time spent on gaming on the internet, time spent on social media, and smoking history were treated as confounding variables. Further statistical testing on the differences of regression coefficients was conducted using a t-test statistic: dividing the difference between the two regression coefficients by the square root of their sum of the squared standard errors. All statistics were tested using two-tailed with significance level set at *p*-value < 0.05.

## 3. Results

Participants (*N* = 300) had a mean (SD) age of 20.89 (1.48) years. Less than half of the participants were male (40.67%), with small proportion of current smokers (0.36%). On average, participants spent 5.29 h (2.34) on smartphones, 3.09 h (1.80) on social networks and 1.19 h (1.53) gaming per day.

Participants had a mean (SD) score of 16.98 (6.48) on the IGDS9-SF, 15.37 (4.11) on the BSMAS, 5.16 (4.47) for Depression; 4.18 (3.83) for Anxiety; 6.72 (4.25) for Stress; and 6.63 (2.14) on the PSQI (Table 1). Additionally, all scores on the IGDS9-SF, BSMAS, DASS-21, and PSQI distributed normally or nearly normally as their skewness values were between 0.34 and 1.15 and kurtosis values were between −0.32 and 2.24. According to the cutoffs on the IGDS9-SF (score of 21 or above) and BSMAS (score of 19 or above), 77 (25.7%) of participants had IGD and 67 (22.3%) had SMA.

Table 2 shows that all the studied variables were significantly correlated (*p* < 0.001). More specifically, IGDS9-SF, BSMAS, depression, anxiety, stress, and PSQI scores were all positively correlated (r = 0.221 to 0.363). The magnitudes of correlations between DASS-21 subscales and IGDS9-SF (r = 0.331 to 0.363) were similar to those between DASS-21 subscales and BSMAS (r = 0.336 to 0.384). Indeed, the Fisher’s r-to-z transformation on dependent samples showed non-significant findings (z = 0.227 to 0.807; *p* = 0.42 to 0.82). Although the correlation between BSMAS and PSQI scores (r = 0.351) showed a numerically larger magnitude than that between IGDS9-SF and PSQI (r = 0.249), the Fisher’s r-to-z transformation on dependent samples showed no significant difference in the correlation comparison (z = 1.509; *p* = 0.13).

Multiple linear regressions in Table 3 (F-values ranged between 6.916 and 10.323; all *p* < 0.001) showed that both IGDS9-SF and BSMAS were associated with all outcome variables (i.e., depression, anxiety, stress, and sleep quality) after controlling for age, gender, time spent on smartphones, time spent on social media, and time spent gaming. As shown by the standardized coefficient (β), IGDS9-SF had a higher association with depression (β = 0.295), anxiety (β = 0.325), and stress (β = 0.339) as compared to BSMAS (β = 0.235 for depression; 0.219 for anxiety; 0.262 for stress) without significance (t = 0.116 to 0.577; *p* = 0.56 to 0.91). In contrast, BSMAS scores had a significantly higher association with poor sleep quality (β = 0.292) as compared to IGDS9-SF scores (β = 0.157; t = 2.436; *p* = 0.01).

## 4. Discussion

This study found that participants with greater IGD and/or SMA severities had more depression, anxiety, and stress. Moreover, participants’ sleep quality was poorer with greater severities of IGD or SMA. According to our Pearson’s correlation and multiple regression results, sleep quality demonstrated a numerically more robust association with SMA severity as compared to IGD severity. On the other hand, similar to findings from Pontes [32], our findings showed that psychological distress demonstrated a numerically more robust association with IGD severity, as compared SMA severity. Although no significant differences were found in the comparisons of Pearson’s correlation coefficients, regression analyses revealed that some relationships were significantly different in the association magnitudes and some were not. Specifically, the sleep quality had a significantly stronger association with SMA severity (β = 0.292) than with IGD severity (β = 0.157); psychological distress had similar magnitudes of association with both SMA severity (β = 0.219 to 0.262) and IGD severity (β = 0.295 to 0.339). We believe that the results of comparing regression coefficients are more robust than those of comparing Pearson’s correlation coefficients, because Pearson’s correlation did not control confounders’ effects, whereas regression analysis did. Therefore, we tentatively conclude that severities of IGD and SMA share similar associations with psychological distress; SMA severity had stronger association with sleep quality than did IGD severity.

Our sample had comparable or higher IGDS9-SF scores (Mean score = 16.98) than those from the general population in Italy (Mean score = 15.93; *n* = 687) [43]. Our sample also had comparable or higher BSMAS scores (Mean score = 15.37) than those from the general population in Italy (Mean score = 14.20; *n* = 734) [48]. When comparing scores to similar ethnic populations, our sample had similar IGDS9-SF scores relative to a sample comprised of young adults from Hong Kong and Taiwan (Mean score = 16.92; *n* = 640) [46] and comparable or slightly higher BSMAS scores (Mean score = 14.46; *n* = 640) [46].

Associations between IGD severity and psychological distress found in the present study are in line with previous studies [2,4,22]. Adolescents with anxiety, depression, or stress may spend excessive time on gaming on the internet as a coping mechanism, to escape from worries and difficulties in reality [22,59]. Unfortunately, gaming may not help people cope with psychological distress in a positive way. Spending more time gaming may provoke more emotional difficulties, and subsequently lead to IGD and related problems [59]. This cycle could further exacerbate symptoms of IGD and psychological distress [59]. Additionally, people with IGD have exhibited relatively less cognitive reappraisal and more expressive suppression, and these tendencies may prevent emotional difficulties from being resolved and the potential for depression to develop or worsen [59].

The present study also found a positive correlation between IGD severity and poor sleep quality, consistent with prior studies. Achab et al. suggested that people with IGD tend to have sleep deprivation or diurnal sleepiness [12], in line with findings that people who engage frequently in gaming are likely to delay their bedtimes and shorten their total time spent in bed [14]. Internet gaming may also arouse central and autonomic nervous systems, which may subsequently lead to increases in sleep latency [20]. Prolonged gaming may lead to physical discomfort, such as muscular pain and headache, which may impact sleep quality [13]. As a result, engaging more frequently in internet gaming may lead to poorer sleep.

The present study found a significantly positive association between SMA severity and psychological distress, in line with previous studies that people with SMA are prone to depressive and anxiety symptoms [4,21]. A sense of loss of control may underlie this phenomenon [9]. Individuals with more problematic smartphone use may experience more functional impairment, and this may interfere with their school/work and family life and eventually lead to increased stress [9]. Individuals with SMA may also be less physically active and have poorer sleep, which may predispose to psychological distress. Social networking provides a platform for persons to escape from negative emotions. However, excessive escapism may disrupt sleep and eventually induce more stress and depressive symptoms [21]. Negative social comparison may also contribute. Feelings of inferiority may be elicited if individuals engage in self-comparison with idealized representations often communicated on social media [4].

The present study found that SMA severity was positively correlated with sleep disturbance. This observation supports prior findings that suggest that time spent on social media is positively associated to sleep problems, such as insomnia, daytime sleepiness, and evening chronotype [13]. A phase delay in the circadian clock could be a reason for the association between SMA severity and sleep quality [13]. Using social media, especially before bed, may lead to difficulties falling asleep and bedtime delay [13]. The relationship between SMA severity and sleep quality may reflect cognitive and emotional arousal when using social media before sleep, with increased arousal preventing sleep [60]. Additionally, blue light from the screen may activate circadian clocks and generate delays in sleep latency [15].

Although both severities of both IGD and SMA were associated with poor sleep, SMA severity was more strongly related. Different strengths of associations could involve multiple possible mechanisms. First, the use of social media may constitute a more on-going process than internet gaming, which may have more well-defined beginnings and endings for each gaming task or component. The sleep patterns of users may be disrupted when they are preoccupied and waiting for replies from others on social media, which may not be well limited in time [14]. Being offline from interactions on social media may generate greater preoccupation and withdrawal when compared to gaming, potentially leading to greater disturbance in sleep quality [35]. Second, different levels and natures of interactions between social media and internet gaming may also explain differences in strengths of associations. Internet gaming may contain similar stimuli within games with main goals involving winning. In contrast, interactions on social media may be more complex, although this may be debatable given the complexities and varieties of games. Individuals may have a variety of goals and aims when using social media, involving multiple interpersonal relations which may require more mental or emotional effort that could lead to poorer sleep. However, as these factors were not assessed in the present study, further investigation is needed to test these presently speculative possibilities.

IGD severity showed associations with psychological distress that were comparable to those between SMA severity and psychological distress. Aspects of gaming and social media warrant consideration in relationships to psychological distress. Internet gaming often involves activities in multi-player chat rooms. Online gamers may thus form a team with individuals they have never physically contacted before when they engage in online games [61]. The teammates may share a goal in the game, and they may discuss their teamwork strategy to win the game. With the mindset of winning and within a virtual competitive environment, these individuals may become aggressive as they focus on winning [61]. Therefore, online gaming may not be an ideal platform for disclosing inner feelings, while social media may be used as a platform to express emotions [62]. Indeed, people with psychological distress can upload a new post on social media to share their current feelings or have an online discussion regarding their work or academic issues in *WhatsApp*. Therefore, the reasons may be different for the associations between IGD/SMA severity and psychological distress; however, both may share similar magnitudes of association with psychological distress. Additional research is needed to examine possible mechanisms linking IGD and SMA severities with psychological distress.

The present findings suggest that teaching faculty and healthcare providers in universities should attend to students’ online gaming behaviors and social media use, especially as IGD and SMA may interact in mutually reinforcing ways [63]. Future research should investigate overlapping and unique factors relating to IGD and SMA. For example, interventions related to SMA may wish to focus on sleeping habits, although given the associations between SMA and IGD with sleep disturbances, the specificity may not be robust.

### Study Limitations and Future Research

This study has several limitations. First, convenience and snowball sampling were used to collect data. Therefore, the representativeness of the collected sample is limited. Specifically, as participants were approached on the basis of availability and questionnaires were distributed to familiar people who may share similar characteristics, the potential bias of self-selection exists and it reduces the representativeness of sample. Thus, generalizability is reduced. Second, the sample size was small, especially as we recruited participants from the general population instead of clinical settings (i.e., those who with diagnosed IGD or SMA); therefore, the findings may not generalize to clinical samples. Third, all study variables (i.e., psychological distress, sleep, and behavioral addiction) were assessed using self-report instruments. Therefore, effects of social desirability or recall biases cannot be excluded. Fourth, the present study cannot make causal inferences given its cross-sectional design. Fifth, we did not explore gender-related differences. Given that males and females may have different propensities for IGD, SMA, and other domains assessed in the present study (e.g., psychological distress that is affective in nature), future studies should systematically examine these relationships in females and males. In such studies, examining motivations (e.g., fear of missing out for SMA) will be important [64]. Lastly, diverse findings in significance testing were observed when we compared the associations between sleep quality and severities of IGD and SMA. Pearson correlation did not support that sleep quality had different levels of associations with severities of IGD and SMA. However, the regression coefficient between sleep quality and SMA severity was significantly stronger than that between sleep quality and IGD severity. Although we believed that the findings from regression analyses are more robust than those from Pearson correlations, future studies are needed to investigate further the robustness of potential differences.

## 5. Conclusions

In conclusion, the present study assessed associations between severities of IGD and SMA and two health outcomes (i.e., psychological health and sleep quality) among Hong Kong university students. The results showed that both severities of IGD and SMA were positively associated with psychological distress and sleep disruption. In addition, although severities of IGD and SMA show similar associations with psychological distress, SMA severity was more strongly associated with poor sleep relative to IGD severity (Table 4). Thus, healthcare providers should consider both domains (psychological distress and sleep) when encountering IGD or SMA, with consideration of the potentially greater impact of SMA on sleep. More research is needed into specific interventions to improve health and sleep in individuals with features of IGD or SMA.

## Figures and Tables

**Table 1:** Participant characteristics.

Characteristic	M ± SD or *n* (%)
Age (year)	20.89 ± 1.48
Gender (Male)	122 (40.67)
Height (cm)	165.57 ± 8.75
Weight (kg)	56.54 ± 10.24
Body mass index (kg/m^2^)	20.52 ± 2.64
Smoke (No) ^1^	279 (99.64)
Monthly income (HKD)	
≤ 1000	34 (12.27)
1001–2000	52 (18.77)
2001–3000	59 (21.30)
3001–5000	88 (31.77)
≥5001	44 (15.88)
Time on smartphones (hours/day)	5.29 ± 2.34
Time spent gaming on the internet (hours/day)	1.19 ± 1.53
Time spent on social media (hours/day)	3.09 ± 1.80
IGDS9-SF score (range: 9–45)	16.98 ± 6.45
BSMAS score (range: 6–30)	15.37 ± 4.11
Depression (range: 0–21)	5.16 ± 4.47
Anxiety (range: 0–21)	4.18 ± 3.83
Stress (range: 0–21)	6.72 ± 4.25
PSQI score (range: 0–21)	6.63 ± 2.14

^1^ With 10 missing values. IGDS9-SF = Internet Gaming Disorder Scale-Short Form; BSMAS = Bergen Social Media Addiction Scale; DASS-21= Depression Anxiety Stress Scales; PSQI = Pittsburgh Sleep Quality Index; HKD = Hong Kong Dollar, 1 HKD ≈ 7.8 UDS. Depression, Anxiety, and Stress were subdomains of the DASS-21.

**Table 2:** Correlation matrix between IGDS9-SF, BSMAS, DASS-21, and PSQI scores.

*r*						
	IGDS9-SF	BSMAS	Depression	Anxiety	Stress	PSQI
IGDS9-SF	--					
BSMAS	0.221	--				
Depression	0.351	0.336	--			
Anxiety	0.363	0.344	0.792	--		
Stress	0.331	0.384	0.792	0.815	--	
PSQI	0.249	0.351	0.351	0.411	0.386	--

IGDS9-SF = Internet Gaming Disorder Scale-Short Form; BSMAS = Bergen Social Media Addiction Scale; DASS-21 = Depression Anxiety Stress Scales; PSQI = Pittsburgh Sleep Quality Index. Depression, anxiety, and stress were subdomains of the DASS-21. All *p*-values < 0.001.

**Table 3:** Regression models assessing associations between problematic use of Internet and health outcomes.

	Coefficient (SE)/Standardized Coefficient			
	Depression	Anxiety	Stress	Sleep Quality
IGDS9-SF score	0.203 (0.047)/0.295 ***	0.188 (0.039)/0.325 ***	0.220 (0.043)/0.339 ***	0.052 (0.023)/0.157 *
BSMAS score	0.250 (0.067)/0.235 ***	0.196 (0.057)/0.219 **	0.264 (0.063)/0.262 ***	0.150 (0.033)/0.292 ***
Age	−0.356 (0.167)/−0.121 *	−0.144 (0.140)/−0.058	−0.171 (0.154)/−0.061	−0.037 (0.082)/−0.026
Gender	0.251 (0.528)/0.028	0.375 (0.444)/0.050	1.030 (0.489)/0.121 *	0.069 (0.259)/0.016
Time on smartphone	0.215 (0.169)/0.102	0.094 (0.143)/0.053	0.125 (0.157)/0.062	−0.021 (0.083)/−0.020
Time gaming on the internet	−0.249 (0.216)/−0.085	−0.165 (0.182)/−0.067	−0.354 (0.200)/−0.128	0.052 (0.106)/0.037
Time on social media	0.039 (0.187)/0.016	0.117 (0.157)/0.057	0.043 (0.173)/0.018	0.121 (0.092)/0.103
Smoking status	1.731 (4.131)/0.023	2.42 (3.479)/0.039	6.44 (3.827)/0.092	−2.055 (2.027)/−0.058
Model summary				
F-value	8.501 ***	8.459 ***	10.323 ***	6.916 ***
R^2^	0.202	0.201	0.235	0.171
Adjusted R^2^	0.178	0.177	0.212	0.146

IGDS9-SF= Internet Gaming Disorder Scale- Short Form; BSMAS= Bergen Social Media Addiction Scale; PSQI= Depression, anxiety, and stress were subdomains of the Depression Anxiety Stress Scales; sleep quality was assessed using Pittsburgh Sleep Quality Index. * *p* < 0.05; ** *p* < 0.01; *** *p* < 0.001.

**Table 4:** Summary of associations between severities of internet gaming disorder (IGD)/social media addiction (SMA) and health measures.

	Depression	Anxiety	Stress	Sleep Quality
Severities of IGD	o	o	o	−
Severities of SMA	o	o	o	+
t (p) ^a^	0.574 (0.57)	0.116 (0.91)	0.577 (0.56)	2.436 (0.01)

+ indicates a stronger association; − indicates a weaker association; o indicates a similar association. ^a^ T-tests that compared unstandardized coefficient of IGD severity on health outcomes and that of SMA severity on health outcomes.
